# The effect of lavender on mood disorders associated with the use of combined oral contraceptives (COCs): a triple-blinded randomized controlled trial

**DOI:** 10.1186/s12906-024-04419-z

**Published:** 2024-03-08

**Authors:** Mina Naghdi, Azizeh Farshbaf-Khalili, Jila Nahaee, Parvin Hakimi, Mahnaz Shahnazi

**Affiliations:** 1 Department of Midwifery, Faculty of Nursing and Midwifery, Tabriz University of Medical Sciences, Tabriz, Iran; 2 Physical Medicine and Rehabilitation Research Centre, Aging Research Institute, Tabriz University of Medical Sciences, Tabriz, Iran; 3 Department of Midwifery, Faculty of Nursing and Midwifery, Tehran Medical Sciences, Islamic Azad University, Tehran, Iran; 4 Women Reproductive Health Research Center, Tabriz University of Medical Sciences, Tabriz, Iran; 5 Department of Midwifery, Faculty of Nursing and Midwifery, Tabriz University of Medical Sciences, Tabriz, Iran

**Keywords:** Contraceptives, Oral, Combined, Mood, Lavandula

## Abstract

**Background:**

The use of contraceptive methods is influenced by their effectiveness, availability, and minimal side effects. OCPs are one of the most effective and widely used methods of pregnancy prevention worldwide. This method not only prevents pregnancy but also helps prevent and treat other diseases. One of the main reasons for discontinuing this method is the emotional disturbances associated with its use. Lavender is an evergreen, fragrant plant that has gained significant attention for its anti-anxiety effects. This study was conducted to investigate the effect of lavender essential oil capsules on mood disorders during the use of COCs.

**Methods:**

This triple-blinded clinical trial was conducted on 60 married women (aged 15–49 years old) who were consumers of COCs, referring to 26 health centers in Tabriz, Iran. The participants were randomly assigned to either the intervention (consuming one gelatin capsule containing 80 mg LEO daily) or control (consuming one placebo capsule daily) group. The intervention continued for 56 days. Scores for positive and negative were determined using the Positive and Negative Affect Schedule (PANAS) questionnaire; and for stress, depression, and anxiety were measured using the DASS-21 questionnaire on day’s 28th and 56th post-intervention. Data analysis was conducted using the t-test and ANOVA with repeated measures, and a p-value of < 0.05 was considered significant for all analyses.

**Results:**

A statistically significant difference was observed in mood disorders, stress, and depression between women receiving LEO or placebo. The consumption of LEO increased the positive mood on day 28 [MD (95% CI): 4.5 (2.1 to 7.0), *p* = 0.001] and day 56 [5.9 (3.4 to 8.3), *p* < 0.001] while decreased the negative mood on day 28 [MD (95% CI): -3.5 (-5.3 to -1.3), *p* < 0.001] and day 56 [-4.3 (-6.3 to -2.2), *p* < 0.001], stress on day 28 [MD (95% CI): -4.9 (-7.1 to -2.8), *p* = 0.001] and day 56 [-5.3 (-7.6 to -3.1), *p* < 0. 001], and depression on day 28 [MD (95% CI): -3.0 (-4.9 to 1.1), *p* = 0.003] and day 56 [-3.1 (-5.0 to 1.2), *p* = 0.002]. There was no statistically significant difference between the two groups in terms of anxiety.

**Conclusions:**

The consumption of LEO with COCs improved mood disorders and reduced stress and depression. The use of hormonal contraceptives and mood changes should be considered by providers. Therefore, regarding the possibility of mood changes, it is expected that appropriate counseling and education will be provided to women who consume COC., providing appropriate solutions, including the simultaneous use of LEO.

**Supplementary Information:**

The online version contains supplementary material available at 10.1186/s12906-024-04419-z.

## Background

Oral contraceptive pills (OCPs) are among the most reliable methods of contraception available. If used correctly, the failure rate of this method is only 0.1%. Globally, around 44% of women aged 15–49 years use modern contraceptive methods, of whom 16% choose OCPs. Based on the latest report by the United Nations (UN), about 11% of Iranian women of reproductive age use OCPs [1]. The advantages of using combined oral contraceptives (COCs) include protective effects against uterine and ovarian cancer, and pelvic inflammatory disease (PID), as well as possible preventive effects against ovarian cysts and iron deficiency anemia. In addition, the return of pregnancy after discontinuing the pill is immediate; their use is under the control of the individual; their use can be stopped at any time without the need for referring to a specialist, and COCs are easily available. Besides, COCs are commonly indicated for treating benign painful gynecological disorders (endometriosis, dysmenorrhea, etc.) [2].

Since the introduction of COCs in the 1960s, several clinical trials and studies have been conducted to investigate their positive and negative effects on women’s physical (menstrual cycle duration, cramps, chest pain, etc.) and psychological (depression, anxiety, mood) health [3, 4]. Recently, researchers have suggested that COCs can be associated with negative effects on mood and psychological health [5]. Mood is an intrinsic feeling affecting almost all aspects of a person’s behavior [6]. Mood disorders manifest as a state of depression that can be caused by various factors, including medications such as COCs, which have been reported to be associated with negative mood disorders [7]. In a study in Sweden, consuming COCs was significantly associated with exaggerated irritability and mood disorders [8].

Regardless of the advantages of using COCs [1], mood problems have been among the common reasons for discontinuing these medications [9]. In a study in the United States, 86% of women consuming COCs either discontinued or changed their medications due to their adverse effects on mood [10]. Mood changes may be the most common perceived side effects of COCs [11]. Multiple mechanisms have been suggested to explain how COCs affect mood. Estrogen acts as a pyridoxine-reducing agent and is involved as a co-factor in some enzymatic reactions. Theoretically, this cofactor can reduce the release of neurotransmitters (such as serotonin (5-HT), 5-hydroxytryptophan, and norepinephrine), which play an important role in regulating behavior [12]. On the other hand, the progesterone existing in COCs, especially second-generation COCs, results in an elevation in mono-oxidase levels at a higher rate compared to natural progesterone, leading to a further decrease in serotonin levels and potentially predisposing to irritability and depression [13].

For years, medicinal plants and their derivatives have been used, either alone or in combination with other drugs, to treat psychological disorders [14–16]. A profusion of natural products can be derived from aromatic and medicinal plants, which have considerable advantages for human health [17]. Due to the side effects of chemical drugs, more people are turning to using medicinal plants for their health [15]. Growing evidence shows that lavender can be effective in the treatment of several neurological and psychological disorders. Studies on animals and humans have confirmed the anti-anxiety, mood-stabilizing, sedative, analgesic, and anticonvulsant effects of lavender [18–20].

Lavender is an evergreen aromatic shrub traditionally used as a cosmetic plant. This plant family includes more than 30 species, dozens of subspecies, and hundreds of hybrids [21]. The main constituents of *L. Angustifolia (Lavandula angustifolia)*, which is the most common lavender species, are linalyl acetate and linalool [22]. The anti-anxiety effects of lavender have been attributed to its volatile linalool [23].

The beneficial effects of lavender essential oil (LEO) have been investigated and compared with several drugs such as lorazepam, paroxetine, imipramine, venlafloxacin, chlordiazepoxide, and diazepam in studies on both humans and animals. In a study on patients with generalized anxiety disorder (GAD), the effects of gelatin capsules containing LEO (Silexan) were reported to be superior to lorazepam in improving anxiety symptoms after six weeks of treatment. Since LEO has zero potential for abuse, it is not associated with withdrawal symptoms, and as suggested in the recent study, Silexan seemed to be more effective than benzodiazepines in relieving general anxiety symptoms [24]. The use of 80 and 160 mg of Silexan (gelatin capsules containing LEO) per day revealed that at the 160 mg dose, Silexan was more effective than paroxetine (20 mg) in the treatment of GAD. Also, Silexan could be safely terminated after ten weeks without causing withdrawal symptoms.

The results of three clinical trials showed that oral administration of LEO (Silexan) at a daily dose of 80 mg improved anxiety, restlessness, and irritability. An investigation reported that the use of Silexan at the dose of 160 mg per day with COCs did not interfere with the effectiveness of COCs [25].

According to our literature search in various databases, no interventional study has been conducted on the effects of LEO on mood disorders in women using COCs for contraception. Clinicians should consider the relationship between the use of hormonal contraception and mood changes. This clinical trial aimed to investigate the effects of gelatin capsules containing LEO on mood disorders associated with the use of COCs in women of reproductive age. Considering the positive effects of lavender on the mood of women in some studies, we hypothesized that if these positive effects are confirmed on COC users, the non-continuation of taking pills may be reduced and mental health will also be improved.

## Methods

### Study design

This study is a two parallel-arm, placebo-controlled superiority trial in which participants, caregivers, and outcome assessors were being blinded carried out from June 5 to December 21, 2022, aiming to explore the effect of LEO on mood disorders in women consuming COCs. This clinical trial compared the means of positive and negative mood scores of mood disorders in women consuming COCs in combination with LEO or a placebo from May to Dec 2022. After being approved by the Ethics Committee of the Tabriz University of Medical Sciences, this trial was registered at the Iranian Registry for Clinical Trials (IRCT). The study was conducted on 60 women referring to comprehensive health centers in Tabriz, Iran, who were recruited by the convenience sampling method.

The COCs used by the participants in this study included levonorgestrel (0.15 mg) and ethinyl estradiol (0.03 mg). Participants in the intervention group daily received gelatin capsules containing 80 mg LEO (L.Angustifolia species: Limonene 13.04%, Linalool 8.96%, Linalyl acetate 35.55%, 1,8 Cineol 37.95%, extracted by hydro-distillation), manufactured by Barij Essence Co. (Batch no. 14,012,121, The quality control of the drug has been carried out by the quality control department of Barij Essance Company, Iran, Tehran) [21] (the plant name has been checked with http://www.theplantlist.org). The placebo was prepared by the same company in the same form (color, weight, shape) as gelatin capsules containing inactive ingredients. The identical LEO and placebo gelatin capsules were provided to the participants according to random allocation and considering the allocation concealment. So, the researchers, participants, and data analyzer were blind in this study.

Random assignment to the study groups was conducted using random blocks with a size of four and six and a ratio of 1:1. The random allocation sequence and packaging preparation were conducted by a person not involved in sample recruitment. The packages included 56 capsules containing LEO or 56 capsules containing the placebo to be used over 56 days. The packages, along with a checklist to mark daily drug consumption and document possible side effects, were provided to the participants by the researcher. In the first face-to-face meeting with the participants, they were fully explained about the drug consumption, study objectives, the benefits and possible side effects of the drugs, and how to complete the checklist. The participants were asked to consume the COCs pills during the first 5 days of menstruation (to ensure that they were not pregnant) once a day after meals. They were also requested to complete the checklist and record any side effects within 30 min after consuming the drug. The participants were instructed to contact the researcher if they suspected pregnancy or wanted to stop taking capsules. The PANAS (Positive and Negative Affect Schedule) and 21-DASS (Depression, Anxiety and Stress Scale − 21 Items) questionnaires were completed to assess changes in the mood, stress, anxiety, and depression scores among the participants on days 28th and 56th after the intervention and compare their scores with the baseline. The questionnaires were completed either by interview, via telephone, or on WhatsApp or Telegram. At the time of completing these questionnaires, the checklists of drug consumption and side effects were also examined. Changes in positive and negative moods were primary outcomes, and changes in depression, stress, and anxiety scores were secondary outcomes in this study.

### Setting and participants

Considering the socio-economic statuses of people in different regions, sample recruitment was conducted in 26 selected healthcare centers (urban, urban-rural, or rural), as well as in the clinics of the Taleghani and Al-Zahra teaching hospitals of Tabriz, Iran. The participants were randomly selected, and their identifications, contact numbers, and medical history (allergies, special diseases, etc.) were extracted from the SIB system (an integrated system for registering health information in Iran). Eligibility criteria for women to enter the study included: age of 15 to 49 years, being married, using COCs as a contraception tool, using COCs for at least the past one month, having a high ratio of negative to positive emotional score based on the PANAS questionnaire (Supp 1), having a registered contact number, using at least one social media platform (WhatsApp, Telegram, etc.) for exchanging photos, questionnaires, etc., having experienced no unpleasant events such as the demise of a family member at least three months before the start of the study, and not suffering from depression (evidenced by a score ≤ 21 in the DASS-21 questionnaire) (Supp 2), not using other conventional methods to control the side effects of COCs, not having allergies to herbal medicines, not being diagnosed with any psychological health problems within the past six months before the study initiation, and not using narcotic, psychotic, or sedative drugs. Accordingly, eligible individuals were contacted by phone and invited to visit the health center if they were willing to participate in the study, and after completing the PANAS and DASS-21 questionnaires and signing an informed consent form by participants or their parent and/or legal guardian (for minors), they entered the study.

### Sample size

Based on a study by Shahnazi et al. [26], and taking into consideration a mean positive mood score of m = 26.05 (SD = 6.27), as well as a 20% elevation in the positive mood score, study power of 80%, and α = 0.05, the sample size was estimated to be *n* = 48. Also, considering a mean negative mood score of m = 32.26 (SD = 6.24), a 20% decrease in the negative mood score, study power of 80%, and α = 0.05, the sample size was calculated as *n* = 24, which was finally chosen as the basis for selecting the number of subjects. Taking into account a 25% rate of drop-out, the final sample size per group was designated as *n* = 30, so 60 people were finally recruited.

### Data collection

The data collection tools included a checklist to record demographic characteristics, a checklist to record possible complications (nausea, indigestion, fatigue, headache, diarrhea, gastritis, sore throat, and nasal congestion), and the PANAS and DASS-21 questionnaires. The PANAS questionnaire was first developed by Watson, Clark, and Tellegen [27], in which 20 items, 10 expressing positive mood and 10 expressing negative mood, are presented in the form of words to assess positive and negative mood states.

The ten items related to positive mood include interest, enthusiasm, strength, eagerness, pride, proudness, consciousness, keenness, persistence, alertness, and liveliness, and the ten negative mood items comprised distress, sadness, guilt, fear, hostility, irritability, shame, restlessness, agitation, and panic. The respondent’s opinion about experiencing each of these feelings in the last week was measured on a 5-point Likert scale, ranging from 1 to 5, and the responses were categorized as (1) zero, (2) low, (3) moderate, (4) high, and (5) very high.

The validity and reliability of this scale have been approved in several studies. Cronbach’s alpha coefficient of this scale was reported as 0.85 in a study by Hosini and Sohraby [28], indicating its acceptable internal consistency. Abul Qasimi [28] also noted that the internal correlation coefficient of the whole scale and its components ranged from 0.74 to 0.94, all of which were statistically significant (*p* < 0.01), denoting the construct validity of the scale. Also, the reliability coefficient of this tool in the report of Abul Qasimi [29] was obtained as 0.65. The validity of the instrument was affirmed by calculating the correlation between positive and negative feelings using tools assessing their related constructs. For example, the consistency of negative mood components was affirmed based on the Hopkins Symptom Checklist (0.72), and the consistency of positive mood components was confirmed using the overt anxiety scale (0.35). Soltanizadeh reported the reliability of this scale as 0.71 [30]. To calculate the total mood score using this questionnaire, the numerical value of each of the subscales was summed up together to calculate the final score. A higher score in each of the subscales shows the presence of a specific type of feeling (positive or negative) in the person.

The DASS-21 questionnaire, developed by Crawford & Henry, is an abridged form of the DASS-42 questionnaire. This scale consists of 21 questions, which are scored on a 4-point Likert scale from 0 (not applicable) to 3 (always applicable). This scale evaluates stress (7 questions), anxiety (7 questions), and depression (7 questions). The levels of stress, anxiety, and depression are then calculated based on the average scores obtained by the individual, where a higher score indicates a more severe condition. The psychometric features of depression, anxiety, and stress have been investigated in several studies. In a study by Henry and Crawford, Cronbach’s alpha coefficients of the whole scale and its three subscales (depression, anxiety, and stress) were reported as 0.93, 0.88, 0.83, and 0.90 respectively [31]. Cronbach’s alpha coefficients of the Persian version of DASS-21 have been reported as 0.85, 0.75, and 0.87 for the depression, anxiety, and stress dimensions, respectively [32].

### Statistical analysis

Data analysis was performed in SPSS 24 software. The mean scores of positive and negative mood, depression, anxiety, and stress were compared between the study groups (after checking for the normality of data distribution) using the independent-t or Mann-Whitney test (before the baseline) and ANOVA with RM adjusted for age and baseline values or Mann-Whitney test (after the intervention). ANCOVA model adjusting for the baseline values and age was used to compare groups in days 28 and 56 after intervention. Within-group comparisons were done using ANOVA with RM, Wilcoxon, and Friedman tests. The significance level for all tests was considered *p* < 0.05.

## Results

### Socio-demographic characteristics

**Fig. 1:**
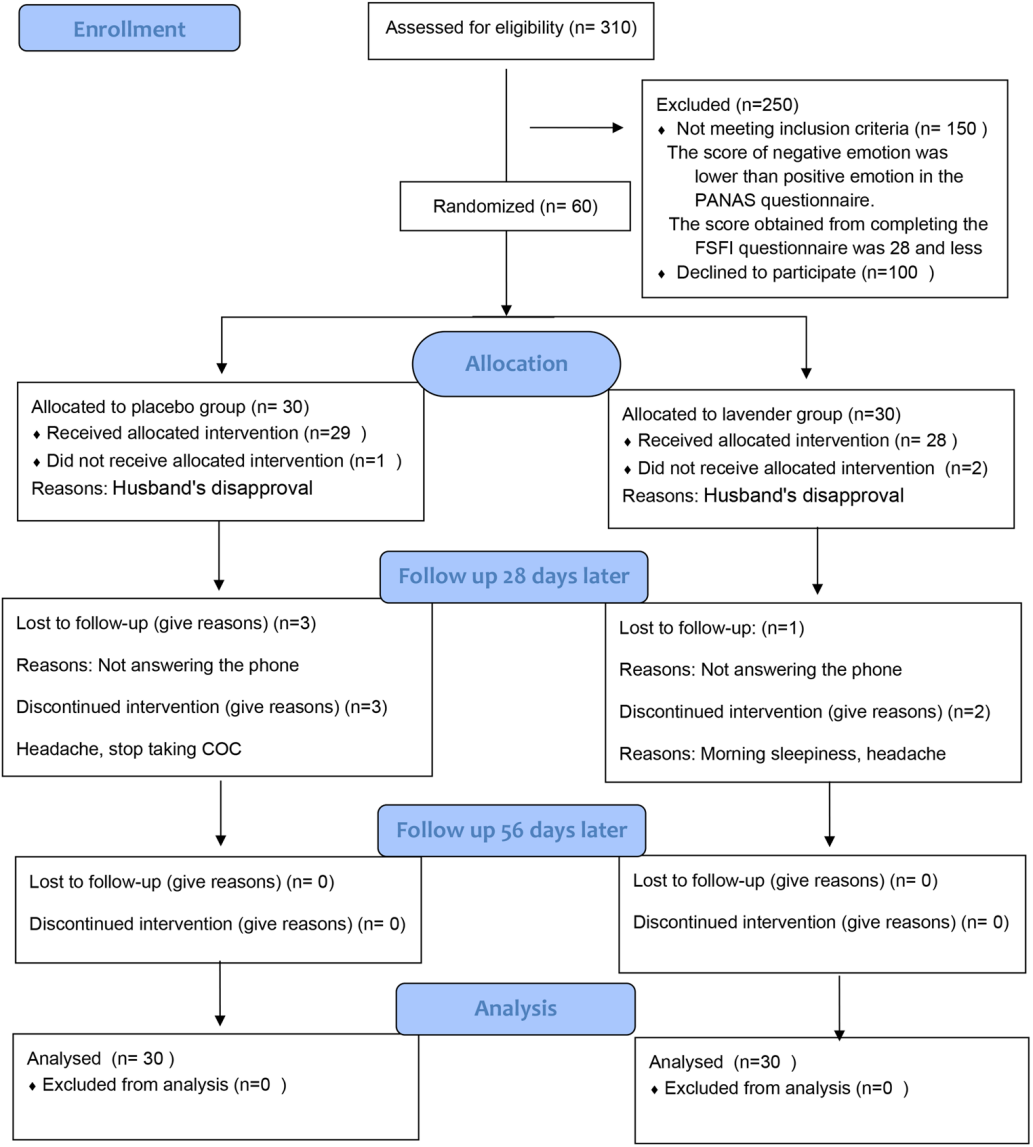
Study procedure flowchart

In this study, 310 women were assessed for eligibility; 150 were excluded for not meeting the eligibility criteria, and 100 individuals refused to participate in the study. Finally, 60 women were randomly allocated into the LEO group, and control groups (30 per group). Five participants in the LEO group and seven in the control group were excluded owing to dissatisfaction or reluctance or side effects (Fig. 1).

Table 1 provides the sociodemographic characteristics of the participants in the two groups of LEO and placebo. Most of the participants in the LEO (73%) and placebo (80%) groups had average income (self-reported). No significant differences were found between the groups in terms of descriptive characteristics (*p* > 0.05) except for age (*p* = 0.035).

**Table 1:** Comparison of the socio-demographic characteristics of the research participants in the two groups receiving lavender and the placebo group

Variables	Lavender group (*n* = 30) n (%)	Placebo group (*n* = 30) n (%)	P
**Age (years)**	32.8^*^ (8.27)	28.83^*^ (5.68)	**0.035** ^**£**^
**Duration of marriage (years)**	9.77^*^ (5.56)	8.1^*^ (4.19)	**0.196** ^**£**^
**Occupation**			**1** ^**&**^
Housewife	24 (80)	24(80)	
working at home	2 (6.7)	3 (10)	
Working outside the home	4 (13.3)	3 (10)	
**Education**			**0.77** ^**&**^
Primary + secondary school and below	12 (40)	9 (30)	
High school	11 (37.7)	10 (33.3)	
Diploma	4 (13.3)	7 (23.3)	
University and above	3 (10)	4 (13.3)	
**Income adequacy**			**0.82** ^**&**^
Yes	3 (10)	2 (6.7)	
No	5 (16.7)	4 (13.3)	
Partially	22 (73.3)	24 (80)	

* Mean (Standard deviation); £: T-test; &: Fisher’s exact test

### Positive and negative moods between groups and within groups

Regarding the positive and negative affect scores before the intervention, no statistically significant difference was observed between the two groups according to the independent t-test.

Within-group comparison (comparing each time point with the baseline) in the LEO and placebo groups based on the ANOVA test showed an increase in the mean positive affect score and a decrease in the mean negative affect score at 28 and 56 days after the intervention compared to the baseline, which was statistically significant in both groups (*p* < 0.001). Also, the repeated-measure ANOVA test showed that there was a statistically significant difference between the three-time points of measurement in both the LEO and placebo groups (*p* < 0.001), showing a significantly more elevation in the mean positive affect score and a significantly less decline in the mean negative affect score over time in the intervention compared to the placebo group.

The means of positive and negative mood scores were compared between the LEO and placebo groups at two separate time points after the intervention using the ANCOVA test and after adjusting for the baseline values and age. The results showed that at 28 and 56 days after the intervention, the mean of the positive mood score was significantly higher in the LEO group compared to the placebo group (*p* < 0.001). Although according to within-group comparisons, the mean positive mood score showed a significant elevation in both the LEO and placebo groups, this elevation was more prominent in the LEO group, resulting in a statistically significant difference between the two groups at 28 and 56 days after the intervention.

Based on the repeated-measure ANOVA test, the increase in the mean positive mood score and the decrease in the mean negative mood were statistically significant compared to the LEO and placebo groups after controlling for the effect of age (*P* < 0.001).

After controlling for the effect of age, comparing the means of positive and negative mood scores between the LEO and placebo groups based on the Greenhouse-Geisser or Sphericity Assumed test (i.e., the interaction of time and group) showed a statistically significant difference between the two groups (*p* < 0.001, Table 2).

**Table 2:** Comparison of positive and negative mood between groups and within groups receiving lavender and Placebo at any point in time and over time

Variable	Lavender			Placebo			Lavender & placebo	
**Positive PANAS**	Mean (SD)	Mean change (95% CI)	P^*^	Mean (SD)	Mean change (95% CI)	P^*^	Mean difference (95% CI)	P^**^
Before	22.7 (3.35)	-		22.36 (3.01)	-		0.33 (-1.98 to 1.31)	0.687^&^
28 days later	30.08 (5.91)	7.38 (5.23 to 9.52)	< 0.001	25 (3.56)	2.63 (1.29 to 3.97)	< 0.001	4.54 (2.06 to 7.02)	0.001
56 days later	30.84 (5.59)	8.14 (6.14 to 10.13)	< 0.001	24.6 (3.94)	2.24 (0.88 to 3.6)	0.002	5.85 (3.43 to 8.26)	< 0.001
P^***^	<0.001			<0.001				
Between the lavender and placebo groups	Mean difference (95% CI)			P^****^			P^*****^	
	3.82 (1.81 to 5.83)			< 0.001			< 0.001	
**Negative PANAS**	Lavender			Placebo			Lavender & placebo	
	Mean (SD)	Mean change (95% CI)	P^*^	Mean (SD)	Mean change (95% CI)	P^*^	Mean difference (95% CI)	P^**^
Before	27.36 (3.12)			26.8 (2.83)			-0.566 (-2.1 to 0.97)	0.465^&^
28 days later	19.64 (3.89)	-7.72 (-9.43 to -6.01)	< 0.001	23.82 (3.33)	-2.97 (-4.44 to -1.5)	< 0.001	-3.5 (-5.33 to -1.68)	< 0.001
56 days later	19.28 (3.86)	-8.08 (-9.77 to -6.4)	< 0.001	23.86 (3.63)	-2.93 (-4.53 to -1.32)	0.001	-4.26 (-6.26 to -2.25)	< 0.001
P^***^	<0.001			<0.001				
Between the lavender and placebo groups	Mean difference (95% CI)			P^****^			P^*****^	
	-2.23 (-3.61 to -0.85)			0.002			0.001	

P^*^: Repeated measure ANOVA (Comparison of each time point with the baseline); P^**^: Between‑group p values after intervention with ANCOVA adjusted for baseline and age (comparison of lavender group and placebo at each time point); P^***^: Within-group repeated measure ANOVA ; P^****^: Between‑group repeated measure ANOVA adjusted for age during the time; P^*****^: Greenhouse-Geisser or Sphericity Assumed test adjusted for age (time and group interaction); SD: Standard deviation; CI: Confidence interval; &: Independent T-test

### Stress, anxiety, and depression scores within groups and between groups

Within-group comparison based on the ANOVA test (comparing post-intervention time points with the baseline) in the LEO group revealed a significant decrease in the average score of stress and depression on days 28th and 56th after the intervention compared to the baseline (*p* < 0.05); however, no significant changes were noticed in these scores in the placebo group at none of the time points. Repeated-measure ANOVA for within-group comparisons revealed that the declining trends in the average scores for stress and depression were statistically significant over time in the LEO group (*p* < 0.05), but not in the placebo group. According to the independent t-test, there was no statistically significant difference between the two groups (LEO and placebo) in terms of the baseline average scores for stress and depression.

After controlling for age and baseline values, and based on the ANCOVA test, a significant decline was observed in the mean scores of stress and depression 28 and 56 days in the intervention compared to the control group after the intervention (*p* < 0.05). Based on the repeated-measure ANOVA test, after controlling the effect of age, the reduction of the mean score of stress in the LEO group (but not the mean score of depression) was observed to be statistically significant compared to the placebo group (*p* = 0.031).

According to the Greenhouse-Geisser or Sphericity Assumed test (i.e., the interaction between time and group) and after adjustment for age, the reductions in the mean scores of stress (*p* < 0.001) and depression (*p* < 0.003) in the LEO group were statistically significant compared to the placebo group.

According to the Wilcoxon test, the mean score of anxiety in the LEO group showed a significant decrease on days 28 and 56 after the intervention compared to the baseline (*p* < 0.05); however, no statistically significant change was observed in the anxiety score in the placebo group.

According to the Mann-Whitney test, there was no statistically significant difference in anxiety scores between the LEO and placebo groups at any time point after the intervention.

According to the Friedman test, the reduction in anxiety over time was statistically significant in the LEO group (*P* = 0.001), but not in the placebo group (Table 3).

**Table 3:** Comparison of stress, anxiety, and depression scores within groups and between groups receiving lavender and placebo at each time point and over time

Variable		lavender			placebo			Lavender and placebo	
		Mean (SD)	Mean change (95% CI)	P^*^	Mean (SD)	Mean change (95% CI)	P^*^	Mean difference (95% CI)	p^**^
**Stress**	Before	12.2 (8.2)	-	-	11.9 (8.2)	-	-	-0.3 (-4.5 to 3.92)	0.876^&^
	28 days later	7.6 (5.9)	-4.6 (-6.7 to-2.5)	< 0.001	12 (6.9)	0.1 (-1.4 to 1.7)	0.845	-4.9 (-7.08 to -2.8)	< 0.001
	56 days later	7.04 (5.7)	-5.2 (-7.6 to -2.8)	< 0.001	12.4 (6.4)	0.5 (-1.3 to 2.3)	0.575	-5.3 (-7.6 to -3.1)	< 0.001
P^***^		< 0.001			0.798				
Between lavender and placebo group		Mean difference (95% CI)			P^****^			P^*****^	
		-3.81 (-7.2 to -0.3)			0.031			< 0.001	
**Depression**	Before	9.8 (6.3)	-	-	8.5 (5.7)	-	-	-1.3 (-4.4 to 1.7)	0.396^&^
	28 days later	6.1 (5.03)	-3.7 (-5.5 to -1.8)	< 0.001	8.3 (4.5)	-0.1 (-1.7 to 1.3)	0.809	-3.04 (-4.9 to -1.1)	0.003
	56 days later	6.2 (4.8)	-3.6 (-5.4 to -1.8)	< 0.001	8.9 (5)	0.4 (-1.02 to 1.8)	0.55	-3.1 (-5.03 to 1.2)	0.002
P^***^		< 0.001			0.594				
Between lavender and placebo group		Mean difference (95% CI)			P^****^			P^*****^	
		-1.41 (-3.96 to 1.31)			0.272			0.003	
		Median (IQR)		p^I^	Median (IQR)		p^I^	p^II^	
**Anxiety**	Before	6 (4 to 10)		-	4 (4 to 8)		-	0.023	
	28 days later	4 (2 to 6)		0.002	4 (2 to 6)		0.399	0.967	
	56 days later	4 ( 2 to 6 )		0.004	4 (2 to 6)		0.5	0.629	
p^III^		0.001			0.206				

P^*^: Repeated measure ANOVA (Comparison of each time point with the baseline); P^**^: Between‑group p values after intervention with ANCOVA adjusted for baseline and age (comparison of lavender group and placebo at each time point); P^***^: Within-group repeated measure ANOVA; P^****^: Between‑group repeated measure ANOVA adjusted for age during the time; P^*****^: Greenhouse-Geisser or Sphericity Assumed test adjusted for age (time and group interaction); p^I^: Wilcoxon test;p^II^: Mann_whitney test; p^III^: Friedman test; SD: Standard deviation; CI: Confidence interval; &: Independent T-test

During the follow-up and after 28 days, two individuals in the intervention group and three people in the control group discontinued taking the capsules due to headaches and dizziness. Since our approach was intention-to-treat, all participants completed the study questionnaires and entered the final analyses. No specific adverse effects were reported by other participants, as evidenced by reviewing the relevant checklist.

## Discussion

The results of this study showed that the use of LEO by COC consumers significantly improved their mood compared to the placebo group and this effect had increased over time. Also, stress and depression significantly declined in the participants using LEO at 28 and 56 days after the intervention compared to the placebo group. Although the level of anxiety also decreased significantly in the LEO group during the follow-up, no statistically significant difference was observed between the LEO and placebo groups.

In a study on 24 healthy young women, Sazawa et al. (2022) compared the effects of aromatherapy cutaneous patches containing lavender applied during sleep for seven nights with that of the placebo on mood and physiological indicators of stress, reporting a significant improvement in the total of score of mood disorders in the intervention group over time [33]. These findings were consistent with our observations in the present study.

The results of a study in Iran showed that the mean scores of sleep quality, life quality, and mood significantly improved in individuals inhaling lavender compared to the placebo group [34], supporting our observation in terms of mood.

Vaziri et al. (2017), in a randomized clinical trial on 56 women, investigated the effects of lavender oil aromatherapy during the early hours after labor on pain, fatigue, and mood one hour and six hours after delivery; the results showed that perineal pain, physical pain, fatigue, and depression gradually and markedly alleviated in the intervention group compared to the control group. After controlling for the interaction between time and group, there was still a significant difference between the two groups, highlighting the efficacy of lavender aromatherapy in the alleviation of postpartum ailments. Moreover, the recent study showed that after adjusting for the interaction between time and group, a significant increase in the mean positive affect score and a significant decline in the mean negative affect score in the lavender aromatherapy group; however, the differences between the two groups were not statistically significant when adjusting for the time-group interaction [20]. This finding was in line with that of the present study as we noticed a significant improvement in both positive and negative affect scores, as well as depression, in the LEO group. However, in the present study, we witnessed a statistically significant decrease in the negative affect score in both groups, which was inconsistent with the report of Vaziri et al. and can be related to women’s psychological changes in the postpartum period. It is noteworthy that our participants consumed COCs for contraception and also since hormonal changes occur physiologically in the woman’s body in the post-partum stage, it can be different from the result of the present study, which was the synthetic hormone used by the woman.

Dong et al. (2016) conducted a study to investigate the effects of an optical-olfactory stimulant on blood pressure and mood in 18-28-year-old people. In this study, light therapy, along with the inhalation of lavender, peppermint, and lemon extracts, was delivered in 5 days The results of the recent study showed that the 5-day administration of the light-olfactory stimulant (i.e., lavender) significantly ameliorated mood disorders in the intervention group compared to the control group [35]; which is in agreement with the results of the present study; but it was not consistent with Shandong’s study in terms of between-group effects (The lack of effect of light and lavender scent alone on mood disorders). A possible reason for this observation could be the different forms of lavender used in these studies (lavender aromatherapy plus light therapy vs. LEO oral supplements). Moreover, the participants in the study of Dong et al. constituted both men and women, but only women participated in the present study. Also, mood assessment indicators measured in the two studies were different, and the duration of follow-up was short in the recent study (i.e., 1, 3, and 5 days) compared to our research (28 and 56 days).

Linalool has been noted to be the most important constituent of lavender, which can act as a sedative by inhibiting glutamate binding [36]. On the other hand, Pennine is another major component of lemon and lavender that has been noted to boost sedative responses in the presence of gamma-aminobutyric acid (GABA) [37].

The results of a clinical trial demonstrated that lavender promotes slight anti-anxiety effects in humans; however, these effects were not observed in patients suffering from severe anxiety [38]. Although the dose of lavender in Bradley et al.‘s study was higher than in the present study, this notion somehow agrees with our observation in the present study, revealing that although anxiety was reduced in the LEO group, the difference in anxiety score reduction was not statistically significant compared to the placebo group.

A review study showed that standardized essential oil extract of *L. angustifolia* (SLO) was well tolerated and effectively alleviated moderate to severe anxiety and depression [39], which was consistent with the results of the present study in terms of a reduction in depression, but inconsistent about anxiety. A possible explanation for the discrepancy observed could be the difference in the type of lavender extract used.

In vitro, studies have been conducted to scrutinize the neuropharmacological mechanisms of essential oils and their chemical ingredients [40]. Studies have delineated the effects of LEO on the central nervous system at the molecular level, indicating an inhibitory effect on the activity of voltage-gated calcium channels [41]. In contrast to some other monoterpenes available in LEO, linalool and linalyl acetate can exert profound inhibitory effects on voltage-gated calcium channels. On the other hand, the active ingredients of LEO have shown affinity toward the N-methyl-D-aspartate (NMDA) receptor and serotonin transporters (SERT). The binding to and inactivating the NMDA receptor and neuronal messages are mostly attributed to linalool and linalyl acetate as linalool can strongly bind to the SERT receptor [42]. On the other hand, a study on rats reported that linalool could hinder glutamate binding in the cerebral cortex [35]. These observations partly explain the molecular function of lavender in alleviating neurological disorders.

Among the strengths of the present study are its triple-blinded design, the small rate of withdrawals during the follow-up, and being a multi-center trial, including clinics covering people with different socio-economic statuses, which boosted the generalizability of our findings.

One of the disadvantages of the present study was the short duration of the intervention (56 days) due to financial restrictions and completing the PANAS questionnaire by self-reporting, which could have affected their responses due to the possibility of different mindsets among the participants. Our suggestion is to explore different doses of lavender and other communities in future research and if possible, it should be done in a longer time.

## Conclusions

The study examined the effect of LEO on mood disorders associated with the use of COCs. The results have shown that simultaneous use of LEO and COCs leads to improvement in a wide range of behavioral changes, including stress and depression. Therefore, appropriate counseling and education should be provided to women using COCs regarding the potential mood changes and appropriate solutions, including the simultaneous use of LEO with the necessity of caution. More interventional studies are needed to reach more conclusive results in this field.

Implications of the study.

It is recommended to provide proper counseling and education to the women using COCs about the possibility of mood changes and offer them appropriate strategies to control these adverse effects, including the attentive consumption of LEO with COCs.

### Electronic supplementary material

Below is the link to the electronic supplementary material.

**Supplementary Material 1**: PANAS Questionnaire

**Supplementary Material 2**: DASS21 Questionnaire

## Data Availability

Data will be made available by the corresponding author on request.

## Abbreviations

COCs: Combined oral contraceptive
LEO: Lavender essential oil
L. Angustifolia: Lavandula angustifolia
PANAS: Positive and Negative Affect Schedule
DASS-21: Depression, Anxiety, and Stress Scale − 21 Items
PID: Pelvic inflammatory disease
GABA: Gamma-aminobutyric acid
NMDA: N-Methyl-D-aspartate
SERT: Serotonin transporters
