# Supplementary material for: The effect of lavender on mood disorders associated with the use of combined oral contraceptives (COCs): a triple-blinded randomized controlled trial
Source: BMC Complement Med Ther. 2024 Mar 8;24:118. doi: 10.1186/s12906-024-04419-z (PMC10921632; doi:10.1186/s12906-024-04419-z)
Supplement: Supplementary file 1 — Supplementary Material 1: PANAS Questionnaire [file 12906_2024_4419_MOESM1_ESM.pdf]

# Positive and Negative Affect Schedule (PANAS-SF)

| Indicate the extent you have felt this way over the past week. | | Very slightly or not at all | A little | Moderately | Quite a bit | Extremely |
|---|---|---|---|---|---|---|
| PANAS 1 | Interested | ☐ 1 | ☐ 2 | ☐ 3 | ☐ 4 | ☐ 5 |
| PANAS 2 | Distressed | ☐ 1 | ☐ 2 | ☐ 3 | ☐ 4 | ☐ 5 |
| PANAS 3 | Excited | ☐ 1 | ☐ 2 | ☐ 3 | ☐ 4 | ☐ 5 |
| PANAS 4 | Upset | ☐ 1 | ☐ 2 | ☐ 3 | ☐ 4 | ☐ 5 |
| PANAS 5 | Strong | ☐ 1 | ☐ 2 | ☐ 3 | ☐ 4 | ☐ 5 |
| PANAS 6 | Guilty | ☐ 1 | ☐ 2 | ☐ 3 | ☐ 4 | ☐ 5 |
| PANAS 7 | Scared | ☐ 1 | ☐ 2 | ☐ 3 | ☐ 4 | ☐ 5 |
| PANAS 8 | Hostile | ☐ 1 | ☐ 2 | ☐ 3 | ☐ 4 | ☐ 5 |
| PANAS 9 | Enthusiastic | ☐ 1 | ☐ 2 | ☐ 3 | ☐ 4 | ☐ 5 |
| PANAS 10 | Proud | ☐ 1 | ☐ 2 | ☐ 3 | ☐ 4 | ☐ 5 |
| PANAS 11 | Irritable | ☐ 1 | ☐ 2 | ☐ 3 | ☐ 4 | ☐ 5 |
| PANAS 12 | Alert | ☐ 1 | ☐ 2 | ☐ 3 | ☐ 4 | ☐ 5 |
| PANAS 13 | Ashamed | ☐ 1 | ☐ 2 | ☐ 3 | ☐ 4 | ☐ 5 |
| PANAS 14 | Inspired | ☐ 1 | ☐ 2 | ☐ 3 | ☐ 4 | ☐ 5 |
| PANAS 15 | Nervous | ☐ 1 | ☐ 2 | ☐ 3 | ☐ 4 | ☐ 5 |
| PANAS 16 | Determined | ☐ 1 | ☐ 2 | ☐ 3 | ☐ 4 | ☐ 5 |
| PANAS 17 | Attentive | ☐ 1 | ☐ 2 | ☐ 3 | ☐ 4 | ☐ 5 |
| PANAS 18 | Jittery | ☐ 1 | ☐ 2 | ☐ 3 | ☐ 4 | ☐ 5 |
| PANAS 19 | Active | ☐ 1 | ☐ 2 | ☐ 3 | ☐ 4 | ☐ 5 |
| PANAS 20 | Afraid | ☐ 1 | ☐ 2 | ☐ 3 | ☐ 4 | ☐ 5 |

***Scoring:***

***Positive Affect Score*:** Add the scores on items 1, 3, 5, 9, 10, 12, 14, 16, 17, and 19. Scores can range from 10 – 50, with higher scores representing higher levels of positive affect.
Mean Scores: 33.3 (SD±*7.2)*

***Negative Affect Score*:** Add the scores on items 2, 4, 6, 7, 8, 11, 13, 15, 18, and 20. Scores can range from 10 – 50, with lower scores representing lower levels of negative affect.
Mean Score: 17.4 (SD ± 6.2)

***Your scores*** on the PANAS: Positive: ____ Negative: ____

Watson, D., Clark, L. A., & Tellegen, A. (1988). Development and validation of brief measures of positive and negative affect: the PANAS scales. *Journal of personality and social psychology*, *54*(6), 1063.
